# Risk Factors and Prognostic Factors in GBC

**DOI:** 10.3390/jcm13144201

**Published:** 2024-07-18

**Authors:** Luiza Tirca, Catalin Savin, Cezar Stroescu, Irina Balescu, Sorin Petrea, Camelia Diaconu, Bogdan Gaspar, Lucian Pop, Valentin Varlas, Adrian Hasegan, Cristina Martac, Ciprian Bolca, Marilena Stoian, Anca Zgura, Gabriel Petre Gorecki, Nicolae Bacalbasa

**Affiliations:** 1 Department of Visceral Surgery, “Carol Davila” University of Medicine and Pharmacy, 050474 Bucharest, Romania; tircaluiza@gmail.com (L.T.); catalinasavin47@gmail.com (C.S.); 2 Department of Surgery, “Carol Davila” University of Medicine and Pharmacy, 050474 Bucharest, Romania; cezarstroescu@yahoo.com (C.S.); sorinpetrea@yahoo.com (S.P.); bogdangaspar@yahoo.com (B.G.); ni-co-lae_bacalbasa@yahoo.ro (N.B.); 3 Department of Visceral Surgery, Center of Excellence in Translational Medicine, “Fundeni” Clinical Institute, 022336 Bucharest, Romania; 4 Department of Surgery, “Ion Cantacuzino” Clinical Hospital, 020026 Bucharest, Romania; 5 Department of Internal Medicine, “Floreasca” Clinical Emergency Hospital, 014453 Bucharest, Romania; came-liadiaconu@yahoo.com; 6 Department of Internal Medicine, “Carol Davila” University of Medicine and Pharmacy, 050474 Bucharest, Romania; marilenastoian@yahoo.com; 7 Department of Visceral Surgery, “Floreasca” Clinical Emergency Hospital, 014453 Bucharest, Romania; 8 Department of Obstetrics and Gynecology, “Carol Davila” University of Medicine and Pharmacy, 050474 Bucharest, Romania; lucianpop@yahoo.com (L.P.); valentinvarlas@yahoo.com (V.V.); 9 Department of Obstetrics and Gynecology, National Institute of Mother and Child Care Alessandrescu-Rusescu, 020395 Bucharest, Romania; 10 Department of Obstetrics and Gynecology, “Filantropia” Clinical Hospital, 011132 Bucharest, Romania; 11 Department of Urology, Sibiu Emergency Hospital, Faculty of Medicine, University of Sibiu, 550024 Sibiu, Romania; adri-anha-segan@yahoo.com; 12 Department of Anesthesiology, Fundeni Clinical Hospital, 022336 Bucharest, Romania; cristina-martac@yahoo.com; 13 Department of Thoracic Surgery, ‘Marius Nasta’ National Institute of Pneumology, 050159 Bucharest, Romania; ciprianbolca@yahoo.com; 14 Department of Thoracic Surgery, Faculty of Medicine and Health Sciences, Sherbrooke University, Sherbrooke, QC J1K 2R1, Canada; 15 Department of Thoracic Surgery, ‘Charles LeMoyne’ Hospital, Longueuil, QC J4K 0A8, Canada; 16 Department of Internal Medicine and Nephrology, Dr Ion Cantacuzino Hospital, 011438 Bucharest, Romania; 17 Department of Medical Oncology, Oncological Institute Prof.Dr.Al.Trestioreanu, 022328 Bucharest, Romania; anca.zgura@umfcd.ro; 18 Department of Medical Oncology, “Carol Davila” University of Medicine and Pharmacy, 050474 Bucharest, Romania; 19 Department of Anesthesia and Intensive Care, CF 2 Clinical Hospital, 014256 Bucharest, Romania; gabriel.gorecki@prof.utm.ro; 20 Department of Anesthesia and Intensive Care, Faculty of Medicine, Titu Maiorescu University, 021251 Bucharest, Romania

**Keywords:** gallbladder cancer, risk factors, prognostic factors

## Abstract

**Background**: Gallbladder cancer (GBC) is a rare entity with a poor prognosis, usually discovered late due to nonspecific symptoms; therefore, over the last years, attention has been focused on identifying the risk factors for developing this malignancy in order to provide an early diagnosis, as well as new prognostic factors in order to modulate the long-term evolution of such cases. The aim of this review is to discuss both major risk factors and prognostic factors in GBC for a better understanding and integration of relevant and currently available information. **Methods**: A literature search was performed using Cochrane Library, PubMed, Google Scholar, Elsevier, and Web of Science; studies published after the year of 2000, in English, were reviewed. **Results**: Over time, risk factors associated with the development of GBC have been identified, which outline the profile of patients with this disease. The most important prognostic factors in GBC remain TNM staging, safety margin, and R0 status, along with perineural invasion and lymphovascular invasion. Both the technique and experience of the surgeons and a pathological examination that ensures final staging are particularly important and increase the chances of survival of the patients. **Conclusions**: improvements in surgical techniques and pathological analyses might provide better and more consistent guidance for medical staff in the management of patients with GBC.

## 1. Introduction

Gallbladder cancer (GBC) ranks 5th among gastrointestinal tract tumors and is the most common malignancy among biliary tract tumors [1]. GBC is an aggressive cancer with no specific symptoms, which makes its discovery late with a poor prognosis and a 5-year survival of less than 5%, especially for advanced stages [2]. Despite advances in chemotherapy, surgery remains the best treatment option for GBC, which can ensure a good prognosis [3]. Histopathologically, gallbladder adenocarcinoma is the most common, in about 70–90% of cases; other subtypes like papillary tumors are found in 5% of cases, while squamous and adenosquamous carcinomas account for 2–10% of cases [4]. In this review, risk factors and the most commonly encountered prognostic factors that influence both the development of GBC and the prognosis of patients with GBC are detailed.

## 2. Materials and Methods

**Figure 1:**
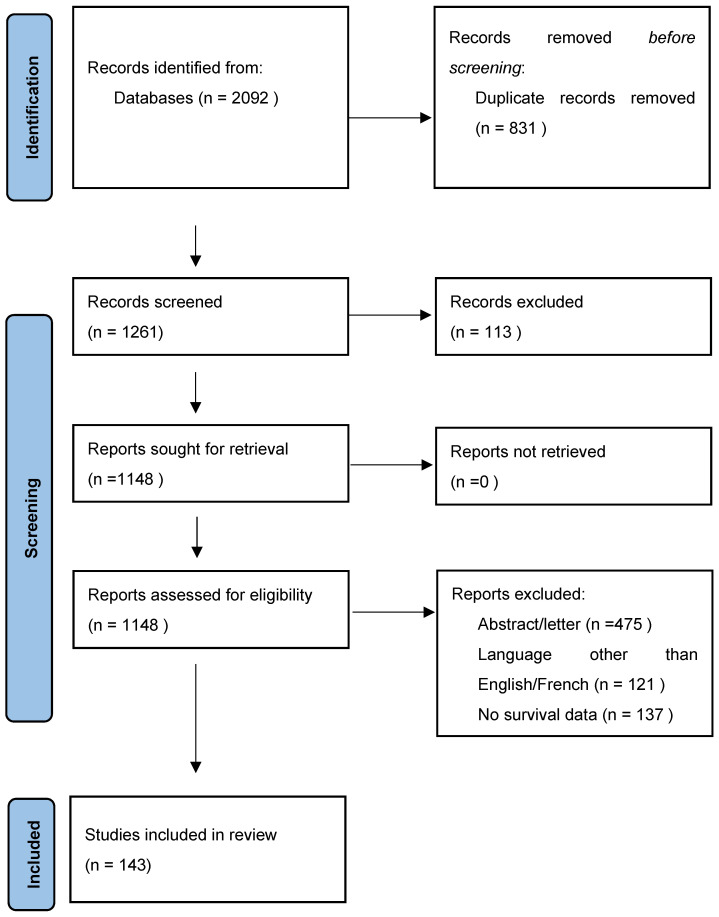
Prisma 2020 Flow diagram.

The PRISMA 2020 guidelines were followed in writing this review. Literature regarding gallbladder cancer was searched in multiple databases: Web of Science, Pubmed, Cochnrane Library, Google Scholar, Embase, Elsevier. The included studies were published between January 2000–January 2024, written in English or French; the research was initially performed in January 2024 and repeated in May 2024, before completing the paper. The keywords that were used both in January and May were: “gallbladder cancer”, “surgery”, “resection”, “prognostic factors”, “invasion”. Three authors—L.T., Ca.S and Ce.S. independently screened the selected articles while the fourth and the fifth—N.B. and I.B. identified and resolved the discrepancies. The final selection of the studies was performed according to the PRISMA 2020 flow diagram presented in Figure 1. Once identified these studies, the following items were analysed: incidence, age, race, gender, anatomical abnormalities, association of cholelithiasis, association of gallbladder polyps, presence of chronic infectious diseases or exposure to toxins such as alcohol, tobacco or aflatoxins. Moreover, prognostic factors such as TNM staging, resection status, lymph node status, distant metastases, perineural and lymphovascular invasion were also taken in discussion and reviewed.

Data was collected in a non-automatical manner by the first author (L.T.) and was assessed by the corresponding author (I.B.).

## 3. Incidence and Mortality

According to GLOBOCAN 2022, the worldwide incidence rate of GBC is 1.2 per 100,000, with the number of newly diagnosed cases in 2022 with this pathology being 122.491; meanwhile, the number of deaths in the same year reached 89.055, which confirms the aggressiveness of the disease. GBC is known to be a cancer that affects women more frequently, and the incidence by gender is 1.4 for women and 0.89 for men. Worldwide, 71.9% of GBC cases are found in Asia, followed by Europe with 10.3%, Latin America 8.5%, Africa 4.5%. Highest incidences are found in Latin America—Bolivia 7.6 per 100.000 and Chile with 5.7 per 100.000, Peru-2.6 per 100.000, as for North America low incidence and mortality rates are reported [5].

Worldwide, 10% of the global GBC burden is attributed to India, with a higher incidence in northern regions (10–22/100,000 population) and lower in South and West India. There has been a steady increase in the incidence in these regions for both males and females [6].

## 4. Risk Factors in GBC

### 4.1. Age and Gender

The incidence of GBC increases with advancing age, particularly in the sixth and seventh decades of life [7,8]. Several studies shown that women are more at risk of GBC [9,10,11,12], being 2 to 6 times more exposed than men [13,14]. This can be explained in part by the high number of pregnancies, multiple births, use of oral contraceptives, hormonal status, out of which estrogen increases cholesterol synthesis and saturates bile content and on the other hand, progesterone slows down the motility of the gallbladder [8,13,14,15], leading to the formation of stones and bile sludge.

### 4.2. GBC and Cholelithiasis

In 1924, Leitch, in an experimental study, blamed the continuous mechanical irritation of the mucosa of the gallbladder due to the presence of stones as being responsible for the development of GBC [16]. Today, it is well known the association between chronic inflammation produced by permanent irritation due to the presence of stones as a pathological condition and the initiation of carcinogenesis, following the metaplasia–dysplasia–carcinoma sequence. The release of inflammatory mediators recruits leukocyte populations, mainly lymphocytes and macrophages, leading to increased reactive oxygen species and COX-2 overexpression. This phenomenon is further followed by deregulation of tumor suppressor proteins and genes, facilitating, therefore, the appearance of mutations with oncogenic potential and cancer progression [17]. The presence of gallstones (GS) is an important risk factor for gallbladder cancer [18]. There are a number of risk factors that are linked to stone formation. These factors can be classified into nonmodifiable factors, such as age, female gender, ethnicity, and genetic predisposition, and modifiable risk factors, such as obesity and diabetes, dyslipidemia, diet, and certain medications [19]. Although 75–90% of patients with GBC have stones [20], less than 3% of patients with stones will develop GBC [8,21,22]. Sizes of the stones greater than 3 cm and a history of GS of over 20 years are associated with a higher risk for GBC due to chronic inflammation and changes occurring in the mucosa [7,21,23]. GS can be successfully detected with an abdominal ultrasound (US), with a specificity of 90%. US is a common method, available at the patient’s bedside, noninvasive, inexpensive, and provides real-time information. US can detect stones as small as 2 mm, and the accuracy of the information obtained being operator dependent. As for computed tomography (CT), it remains inferior to ultrasound due to the fact that quite a large proportion of stones are iso-attenuated in relation to the surrounding bile, remaining invisible to CTt. Magnetic resonance imaging (MRI) is another method in effective stone detection but at a higher cost compared to CT or US. One imaging method does not exclude another, and other features and complications of gallstones, such as cholecystitis, pancreatitis, stones in the common bile duct, or associated tumor processes, can be identified [24].

Porcelain gallbladder (PG), the last stage of chronic cholecystitis, is gallbladder calcification with thickening of the walls; in 95% of cases it is associated with cholelithiasis, and most patients are asymptomatic [25], with the estimated incidence of PG being less than 1% [26]. GBC develops because of chronic inflammation of the mucosa in patients with PG [26,27,28]. There is a difference in the reported incidence of GBC associated with PG in the reviewed literature, with some older papers reporting an incidence between 12.5 and 61%. Calomino et al. performed a study on this subject, with 1050 cases and reports, and there was an incidence of GBC in 2.6% of cases. Regarding the surgical treatment of PG, opinions are divided; some authors recommend laparoscopic cholecystectomy for uncomplicated PG and, in contrast, for complicated PG open cholecystectomy is preferred. Whenever GBC is suspected in a patient with PG the open approach is indicated to avoid eventual tumor dissemination [25]. On the other hand some authors do not indicate prophylactic cholecystectomy, unless there are specific indications of gallbladder disease.

### 4.3. Biliopancreatic Ductal Junction Abnormalities

Other important risk factors for GBC are biliopancreatic ductal junction abnormalities (BPMs), a congenital condition in which the bile duct and pancreatic duct join outside the duodenal wall, resulting in a long common duct, remaining outside the direct action of the Oddi sphincter [29]. In 1906, Arnolds described for the first time, in an autopsy case, biliopancreatic ductal junction abnormality and congenital dilation of the bile duct [30]. The literature describes BPMs with or without congenital bile duct dilation, and patients with associated dilation are more often symptomatic, with the most common symptoms being abdominal pain, jaundice, vomiting, or fever [31,32]. In the process of carcinogenesis, the reflux of pancreatic juice, higher pancreatic duct pressure, and biliary stasis play important roles, leading to a chronic inflammatory process (similar to that generated by stones), which induces the activation of the hyperplasia–dysplasia–carcinoma sequence [32]. In most cases, high levels of amylase are found in the bile duct or in the gallbladder [29,33,34]. Patients with BPMs are at increased risk of developing biliary tract cancers, and those without associated common bile duct dilation more often develop GBC. A BPM can be diagnosed by endoscopic retrograde cholangiopancreatography (ERCP), percutaneous transhepatic cholangiography (PTC), magnetic resonance cholangiopancreatography (MRCP), three-dimensional drip infusion cholangiography computed tomography (3D-DIC-CT), or endoscopic ultrasonography (EUS) [35].

Deng et al. [36] performed a meta-analysis to see if there is an association between BPMs and GBC. The results showed that patients with BPMs but without common bile duct dilation more frequently developed GBC, and a profile of younger females without gallbladder stones was identified.

A nationwide Japanese survey comprising 2651 adult patients with BPMs encountered GBC in 37.4% of patients without dilation and in 13.4% with dilation, compared to extrahepatic cholangiocarcinoma which was found in 3.1% and 6.9% of cases, respectively [33]. Kamisawa et al. [37] analyzed a series of 145 patients with BPMs, 73 patients with and 72 patients without dilation. They found that GBC occurred in 68% of patients without associated dilation and in 21% of cases with dilation.

In 2012, the Japanese Study Group on Pancreaticobiliary Maljunction in collaboration with the Japan Biliary Association (JBA) created clinical practice guidelines regarding the approach and management of BPMs. For patients with a BPM but without congenital common bile duct dilation, prophylactic cholecystectomy is indicated to prevent GBC, whether they are symptomatic or not [31].

The higher incidence of GBC compared to biliary tract cancer in patients with BPMs may also be explained by damage to gallbladder mucosa due to chronic inflammation from reflux and stasis. High γ-H2AX expression, as a marker of DNA degradation and the failure of its repair response, along with DNA double-strand break formation, was found in the biliary tracts of patients with BPMs and at even higher concentrations in patients with BPMs and cancer at the same time. DNA double-strand breaks are recognized in the carcinogenesis process and considered markers of DNA damage. Kuiraishi et al. [38] observed that γ-H2AX expression is even stronger in patients with BPMs without dilation compared to those with dilation, with the median positive proportions being 17.3% and 9.7%, respectively. Kamakura et al. [39] associated mucosal lesions with high levels of γ-H2AX expression, particularly in the gallbladder, in patients with BPMs; meanwhile, it seems that biopsies could be used to predict GBC and bile duct cancer, while γ-H2AX may have a role in early cancer detection as a prognostic indicator in patients with BPMs. In conclusion, a BPM is a high-risk congenital malformation for GBC, and a practical strategy for early detection is needed.

Also, occult pancreatic juice reflux can lead to GBC. In these cases, the pancreaticobiliary junction is anatomically normal, and it causes high amylase levels in the bile duct and in a gallbladder associated with hyperplastic and dysplastic mucosal lesions, leading to gallbladder carcinoma [40,41,42]. Although the mechanism for occult reflux remains unclear, a possible cause could be dysfunction of the Oddi sphincter, such as stenosis or dyskinesia. Occult reflux incidence was found in 36.6% of patients with benign biliary disease; 27.7% of cases had associated cholangiocarcinoma, and 62.5% of cases had GBC [40].

### 4.4. Gallbladder Polyps

Gallbladder polyps (GPs) are benign lesions arising from the mucosa; most are asymptomatic and found incidentally on routine abdominal ultrasound [19]. Detection of polyps is increasingly common due to the expansion of imaging investigations and their use. In general, patients with polyps ≥ 10 mm are referred to the surgical service for prophylactic cholecystectomy, preventing malignant transformation. The worldwide prevalence rate of polyps in adults is between 3%and 7% [43], and the prevalence rate of polyps found on cholecystectomy specimens is between 2 and 12% [44]. Christensen and Ishak, in 1970, classified GPs into benign and malignant polyps [43]. Benign polyps are divided into neoplastic and non-neoplastic polyps; the most common non-neoplastic polyps are cholesterol polyps at 60–70% [44], and for the neoplastic ones, the most common are gallbladder adenomas at 1–5% [45].

The European Joint Guidelines, which were first developed in 2017 and then updated starting in May 2020, currently recommend cholecystectomy for the following: sessile polyps, polyps ≥ 10 mm, polyp growth on follow-up up to 10 mm, or associated symptoms. Cholecystectomy is also recommended for patients with polyps who have associated risk factors, such as age over 60, primary sclerosing cholangitis, or Asian ethnicity. For polyps between 6 and 9 mm, ultrasound follow-up is recommended at 6 months, 1 year, and 2 years; for polyps below 5 mm and without associated risk factors, follow-up is not necessary [46].

The Society of Radiologists in Ultrasound provides recommendations based on risk stratification using information provided by abdominal ultrasound, namely, morphological features such as size, type of polyp (sessile vs. pedunculated), and gallbladder wall thickness. On the basis of these, GPs are stratified into the following three categories: extremely low risk, low risk, and indeterminate risk [47]. Similarly, but with more sonographic features included (size, shape, number of GPs, the appearance of the gallbladder wall, vascularity, and the presence of stones or sludge), Tang et al. proposed another stratification model of malignancy risk for incidentally detected polyps to facilitate clinical management and determined the following four groups: extreme low risk, low risk, intermediate risk, high risk. Single oblate or round GPs larger than 12 mm, polyp vascularity, and presence of gallbladder stones or sludge were considered risk factors for neoplastic polyps. Malignant polyps, however, were found to have the following characteristics: size ≥ 14 mm, oblate shape, single, disrupted gallbladder wall, and presence of gallbladder stones or sludge. For the high-risk group, malignant polyps were identified in 70.37% of cases [48].

Onda et al. [49] presented, as strong factors for a polyp to undergo malignant transformation, age ≥ 65 years and size ≥ 13 mm but also the presence of stones, solitary polyps, and sessile polyps.

Furthermore, a comprehensive meta-analysis of more than 67,000 patients with GPs detected on abdominal ultrasounds showed that the risk of malignant transformation was lower than previously reported and extremely low for polyps smaller than 10 mm [50].

Another retrospective study conducted on 622,227 adults over a 20-year period showed that polyps were detected by ultrasonography in 35,856 (5.8%) patients, out of which only 19 adults developed GBC. GBC development was almost never associated with initially detected polyps under 10 mm and rarely associated with polyps of 10 mm or larger. Because of the results obtained, the authors of this study suggest that the 10 mm limit must be modified [51]. Also, in patients with primary sclerosing cholangitis, a disease associated with hepatobiliary cancers, the American College of Gastroenterology’s clinical guidelines lower the limit of the polyp size to 8 mm for the indication of prophylactic cholecystectomy and recommend annual ultrasound surveillance [52], while the European and American Associations for the Study of Liver Diseases recommend in their guidelines a cholecystectomy regardless of the polyp size [53].

### 4.5. Chronic Infections

Another risk factor is that of pathogen infections, which cause mucosal damage through chronic or acute inflammatory responses and lead to activation of the hyperplasia–displasia–carcinoma sequence. Helicobacter pylori, a Gram-negative germ, is associated with gastric cancer and peptic ulcers but also with cholelithiasis, cholecystitis, and biliary tract cancers, including GBC, and H. pylori DNA has been isolated from tissue samples of patients with GBC [54]. It is believed that H. pylori reaches the biliary tree via a possible translocation, via portal circulation, or by the presence of bile reflux through the Oddi sphincter. As a result, chronic inflammation occurs and leads over time to stone formation, another stressor for the gallbladder mucosa, participating in the development of GBC [55,56]. Different species of Helicobacter, including H. pylori, H. spp., H. bilis, and H. hepaticus, isolated from bile samples are associated with a three-fold increased risk of developing biliary tract malignancies [57]. Salmonella typhi and Para typhi, which are responsible for typhoid and paratyphoid fevers, are found in endemic areas such as India where the incidence of GBC is high. The associated relationship between Salmonella and GBC is mainly recognized for the generated chronic inflammation, damaging the gallbladder mucosa. Through chronic exposure Salmonella typhi produces certain carcinogenic compounds involved in the development of GBC, and antibodies to Salmonella have been found in patients with GBC, especially in men and patients with GS [58]. Given how Salmonella is harbored in GS, the effect of this risk factor is twofold, as the surface of GS enhances colonization, making it more difficult to treat [6].

### 4.6. Obesity

Obesity is a public health problem, and it is well known that obesity is associated with the risk of cardiovascular disease, diabetes, formation of gallstones, and certain cancers. Some studies show that obesity increases the risk of GBC, especially among women [59], and for every 1 kg/m^2^ of BMI, the risk of developing GBC increases by 4%; consequently, for overweight people, the risk is 10% higher, and for obese people, the risk reaches 58% [60]. One possible explanation for why obesity increases the risk of GBC among women would be due to hormonal status, mainly estrogen, which promotes the retention of cholesterol in the bile, and the higher the weight, the higher the risk of forming stones, and, therefore, the risk of GBC also increases [61]. Therefore, preventive measures and population education should be introduced to promote physical activity, low sugar consumption, low-carbohydrate diets, and the adoption of a healthier lifestyle [62].

### 4.7. Alcohol Consumption and Tobacco

Alcohol and tobacco use have historically been associated with the risk of developing certain cancers, but the data and evidence for the risk of developing GBC among people who smoke and drink alcohol are not strong. Wenbin et al. [63] conducted a meta-analysis on smoking and gallbladder cancer and concluded that smokers have a 45% higher risk of developing GBC than nonsmokers, regardless of alcohol consumption and the presence of gallstones. In contrast, McGee et al. [64] extracted data from 26 retrospective studies and analyzed the risk of developing cancer in the biliary tract; they demonstrated that smoking has been associated with malignancies of the ampulla of Vater and intra- and extrahepatic bile duct neoplasms. There was no convincing evidence that smoking is a risk factor for GBC. The same article analyzed the role of alcohol consumption as a risk factor for intrahepatic and extrahepatic biliary tract neoplasms but with no clear evidence that it increases the risk of GB or ampullary neoplasms. Alcohol consumption was quantified as drinks per day, where one drink consisted of 14 g of ethanol. Therefore, before drawing any conclusions, further studies are needed to clearly establish a relationship between alcohol, tobacco consumption, and GBC.

### 4.8. Exposure to Chemical Elements and Compounds

Aflatoxins (AFs) are a group of secondary metabolites produced by Aspergillus parasiticus, Aspergillus flavus, and, more rarely, Aspergillus nomius, fungi that are found in many agricultural products, cereals, dried fruits, nuts, spices, oilseeds, etc., for which these AFs have an affinity. Mycotoxins were discovered in the 1960s when several domestic birds died from feeding on Brazilian peanut flour. So far, almost 20 types of AFs have been discovered, with the following four being the most important: AFB1, AFB2, AFG1, and AFG2. Using chromatography techniques, they have been characterized as blue B1 and -2 and green G1 and -2 [65].

As The International Agency for Research on Cancer (IARC) classifies AFs as a Group 1 carcinogenic and mutagenic risk, chronic exposure to only small amounts of AFs should be considered for the health of the individual. Given that an association between AFs and liver cancer has been demonstrated, AFs could cause other cancers too, such as GBC, due to excretion into bile, with the accumulation of AFs in the biliary tract [66]. Koshiol et al. tested the association between AFs and GBC, and in a study of 84 patients with GBC, they found AFB1 in 50% of GBC cases [67].

High levels of AF were also detected in red chili peppers from Chile, Bolivia, and Peru, countries with a high incidence of GBC [68,69], and AFs may play roles in the development of GBC. Another mycotoxin that has an affinity for grains, spices, and seeds is Ochratoxin A (OA), produced by Penicillinum and Aspergilus, and high levels were found in the South American countries of Chile, Bolivia, and Peru. Ikoma et al. found high levels of OA in dried red chili peppers compared to fresh chili peppers, in which no high concentrations were found. The authors suggest that this mycotoxin may have a role in the development of GBC. Postharvest conditions are the most important, as this mycotoxin has not been detected in large amounts in fresh chili peppers [70].

Arsenic (As), according to the IARC, is classified as carcinogenic to humans, and the World Health Organization (WHO) ranks it among the top 10 chemicals of public health concern. High concentrations of As are found in groundwater in Chile, Argentina, Bangladesh, Mexico, India, China, USA, and Vietnam, and they largely coincide with the high incidences of GBC in these countries. The sources of arsenic exposure are mainly in water, such as drinking water, crop irrigation water, and food preparation water. The WHO recommended an As limit for drinking water of 10 microg/L [71]. Lately, increasing evidence shows that there is an association between As and the risk of developing GBC. In Bihar, India, near the Ganges River, a region with a high incidence of GBC, a large proportion of the population has arsenic poisoning due to groundwater pollution and chronic exposure, and high levels of As have been identified in the hair, blood, stones, and gallbladder tissues of patients with GBC [72]. In Chile, a small cohort study comprising 35 patients with GBC and 42 patients without GBC, found high As levels in 5 patients with GBC (14%), and all 5 patients were female. Elevated concentrations of As were not found in non-GBC cases [73].

In addition, in a large population study, other heavy metals have been associated with GBC risk, such as cadmium, vanadium, chromium, copper, and molybdenum. Metals found in patients with gallstones, such as arsenic, boron, lithium, and molybdenum, are being recognized as an important risk factor in their progression to GBC [74].

## 5. Prognostic Factors in GBC

Currently, surgical treatment of GBC remains the only option to achieve long-term results and is an important prognostic factor in GBC. The surgical approach depends on the preoperative staging of GBC and ranges from simple cholecystectomy to extended cholecystectomy with liver resection, major liver resection, main bile duct resection, or extended resection to surrounding organs. Important prognostic factors in GBC that have an impact on long-term survival are surgery type, TNM score, tumor grading, perineural invasion, lymphovascular invasion, and R0 status. Few studies are specifically done for each item, and most of the information comes from retrospective studies analyzing clinicopathological characteristics of patients with GBC.

### 5.1. TNM Staging

Worldwide, the most widely used cancer staging system is that of the American Joint Committee on Cancer (AJCC), which combines the efforts of both the AJCC and International Cancer Control (UICC) and uses the TNM score, which looks at the size and extent of the primary tumor—T, the involvement of regional nodes—N, and the absence or presence of distant metastases—M. Compared to the 7th edition, the 8th edition of the AJCC (Table 1) brings new modifications and subclassifies T2 into T2a—tumor invades perimuscular connective tissue on the peritoneal side and T2b—tumor invades perimuscular connective tissue on the hepatic side. For T2b, a lower survival has been reported compared to T2a. Modifications were also made for the N category, and the staging of N is changed from the location of the positive nodes to their number, so that N1 means positive nodes between 1 and 3, N2 means ≥ 4 positive regional nodes, and to be able to establish N it is necessary that a minimum of six nodes are being analyzed by the pathologist. Also, N2 from the 7th edition becomes M1 in the 8th edition, so the celiac, peripancreatic, and superior mesenteric nodes are framed as M1, along with other distant metastatic sites [75]. TNM is the strongest prognostic factor in GBC and can tip the balance in terms of survival one way or another.

### 5.2. T1 GBC

Many authors have demonstrated over time that for Tis/T1a GBC, a simple laparoscopic or open cholecystectomy is sufficient and 5-year survival is almost 100%. But for T1b, things are not so clear, and there are debates about simple cholecystectomy vs. extended cholecystectomy. The National Comprehensive Cancer Network (NCCN)’s guidelines recommend T1b radical cholecystectomy and regional lymphadenectomy [76]. In contrast, the Japanese guidelines recommend simple cholecystectomy, with T1b treated as a local disease, reaching down to the muscle layer [77]. Lee et al. conducted a systematic review and showed that there is no conclusive evidence that an extended cholecystectomy for T1b is superior to simple cholecystectomy, but considering that in one study lymph node involvement was reported in 11% of cases, they still recommend lymphadenectomy [78]. Kim et al. showed in an international multicenter study that a simple cholecystectomy is similar in terms of survival to that offered by an extended cholecystectomy and the reported 5-year survival is 93.7% vs. 95.5% [79].

In contrast, Vo et al. [80] performed a retrospective study with 464 patients, with 247 submitted for a simple cholecystectomy and 217 submitted for a radical cholecystectomy en-bloc with hepatic resection and regional lymphadenectomy according to the NCCN guidelines. Positive margins were detected in 6.1% of patients who underwent a simple cholecystectomy and 2.3% of patients who underwent a radical cholecystectomy and regional lymphadenectomy. Of patients who underwent radical cholecystectomy and regional lymphadenectomy, 14.8% had positive nodes and 83.4% had negative nodal status; for the remaining 1.8% of patients, the nodal status could not be assessed. Adjuvant therapy was administrated in 53.1% of cases with positive nodes, in 9.4% of cases with negative nodes, and, respectively, in 10.9% of cases that had previously been submitted for simple cholecystectomy. Patients in the latter case who received adjuvant therapy had positive margins and poor or undifferentiated tumor differentiation. Survival at 5 years was better for those who underwent radical cholecystectomy and regional lymphadenectomy, at 57.7% vs. 48.3% for patients who underwent simple cholecystectomy. This study concludes that for T1b patients, radical cholecystectomy and regional lymphadenectomy are indicated and adherence to existing guidelines should be higher.

As studies show different results for T1b GBC and there is still debate concerning the surgical approach in T1b (i.e., simple cholecystectomy or extended cholecystectomy), Wang et al. [81] set out to clarify this situation by finding if there is any association between tumor size and lymph node metastasis and whether this can dictate the surgical technique. A total of 277 patients were enrolled in this study, out of which 127 patients received a lymphadenectomy. From these patients, 23 had a tumor ≤ 1 cm and no lymph node metastases, and 104 patients had a tumor ≥ 1 cm, 15 of which had lymph node metastases present. In the group with a tumor ≤ 1 cm, no survival differences were observed in terms of simple cholecystectomy vs. extended cholecystectomy compared to the group with a tumor ≥ 1 cm; consequently, the authors recommend a simple cholecystectomy for T1b tumors ≤ 1 cm and an extended cholecystectomy for those ≥ 1 cm.

The multicentric, retrospective Operative Management of Gallbladder Cancer (OMEGA) cohort study included 3676 patients who underwent GBC resection, across 133 centers, and showed that overall survival and recurrence-free survival were related to T stage, N status, and resection margin. Regarding liver resection for T1b, the authors of this study find no improvement in survival but recommend regional lymphadenectomy [82].

Recently, Goel et al. [2] concluded that for T1b patients the positive lymph node rate was 21%, and it is necessary to include lymphadenectomy as a standard of care for better staging. Ren et al. reached the same conclusion and obtained better survival for patients undergoing cholecystectomy and lymphadenectomy. Patients who underwent hepatectomy had no improvement in survival [83]. In conclusion, even if there are difficulties in deciding which is the best surgical approach for T1b, a rigorous preoperative staging is necessary so that patients are not underdiagnosed and left with incomplete treatment.

### 5.3. T2 GBC

For T2 GBC, an extended cholecystectomy en-bloc with wedge hepatic resection of about 2–3 cm or hepatectomy of segments IVb-V is recommended, together with regional lymphadenectomy, as follow: cystic duct nodes, common hepatic duct nodes, and hepatoduodenal ligament nodes, i.e., hepatic artery and portal vein nodes and upper posterior pancreaticoduodenal nodes [84].

Shindoh et al. [85] gathered 437 patients from four high-volume centers in North and South Americas, Asia, and Europe in a study and analyzed whether there is a correlation between tumor location and long-term survival. They divided the tumors into liver-sided and peritoneal-sided tumors. Liver-sided tumors correlated strongly with a higher incidence of vascular invasion, lymph node invasion, and liver metastases; consequently, they reported poorer survival than cases with peritoneal-sided tumors, with the 5-year survival rate being 76% vs. less than 50%. Adjuvant chemotherapy is also suggested for T2 hepatic-sided tumors even if patients have undergone curative surgery. In this respect, it is considered that all T2s should benefit from cholecystectomy with liver resection and lymphadenectomy for good survival outcomes.

Following the T2 subclassification introduced by the AJCC, several authors have conducted numerous studies over time in an attempt to validate the new classification and to analyze whether there are differences between T2a (i.e., peritoneal side) and T2b (i.e., liver side) tumors regarding surgical strategy and long-term outcome, including survival and recurrence.

Kang et al. [86] published a meta-analysis to determine the prognostic significance in patients with a GBC location according to the AJCC subclassifications T2a and T2b and to assess which is the optimal surgical option for each location. For this meta-analysis, seven studies met all inclusion criteria, with a total of 1789 patients. Overall survival at 5 years for T2b was significantly worse than for the T2a group, with a 76–40% rate for T2b vs. 96–60% for T2a, this being the range between the highest and lowest survivals from the included studies. The lymph node metastasis rate for T2b was 36.6% vs. 26.6% for T2a. Overall, perineural invasion and vascular and lymphatic invasions occurred more frequently in the T2b group compared to the T2a group. A trend of better survival was recorded for extended cholecystectomy vs. simple cholecystectomy but without statistical significance, and this is partly because some papers did not use standard surgical techniques of resection terms to describe the operations they performed. For example, in some studies “simple cholecystectomy” actually included lymph node dissection. This meta-analysis shows that T2b is a worse prognostic factor in T2 GBC.

In the same period, Alrawashdeh et al. [87] conducted a systematic review and meta-analysis consisting of 15 retrospective studies and 2531 patients to see the differences in survival between T2a and T2b. In terms of the surgical approach, liver resection data were extracted from nine studies; therefore, 81% of T2a patients and 79% of T2b patients were submitted for liver resection, including wedge resection, segmental hepatectomy, or hemihepatectomy. In seven studies with similar data and analysis, 90% of T2a patients and 88% of T2b patients received lymph node dissection. In six studies that provided data regarding relapse, cases with T2b tumors had a significantly higher risk than those with T2a; meanwhile, seven studies included data regarding the administration of adjuvant chemotherapy and demonstrated that T2b patients received, more frequently, adjuvant therapy than T2a patients. Overall survival was reported in nine studies; T2b patients had poorer survival than T2a patients, and six studies analyzed individual patient data and found that survivals at 5 and 10 years for T2a were 74.2% and 71.8%, respectively, and for T2b 59.9% and 53.6%. Overall, this meta-analysis confirms the results obtained by other authors: T2a patients reported a better overall survival than T2b patients. Also, the authors of this meta-analysis showed that all T2 patients who underwent liver resection were associated with better 5-year survival than those without liver resection, at 82.9% vs. 59.3%. A recent meta-analysis by Matsui et al. [88] shows that patients with T2 GBC tumors who underwent wedge resection had better postoperative outcomes than those with IVb–V liver segment resection in terms of postoperative complications. Patients who had IVb–V liver segment resection had superior oncological outcomes compared to wedge resection, and the latter is a good technique as long as the resection margins are negative. One explanation for the better results with IVb–V liver segment resection may be that it rules out possible occult liver metastases, since the cystic vein directs flow through the veins of segments IVb and V.

In conclusion, for a better long-term survival, lymphadenectomy and liver resection should be performed for T2 tumors, also standardization of the definitions for liver resection should be conducted to facilitate the work of researchers and to achieve more concrete results, and, as in cases of T1b, rigorous preoperative staging is necessary.

### 5.4. T3–T4 GBC

An advanced T stage does not preclude curative-targeted surgery and, frequently, multi-organ resections are involved as long as R0 resection is feasible.

For T3 cases, en-bloc cholecystectomy with liver bed resection or hepatectomy segments IVb–V and lymph node dissection, including cystic lymph node, main bile duct nodes, hepatoduodenal ligament, and upper posterior pancreaticoduodenal nodes, is recommended.

For T4 tumors, to obtain cancer-free margins, extensive resections are required, such as major liver resections with more than three segments, pancreatoduodenectomy, main bile duct resection, colon resections, or gastrectomies, which are associated with postoperative complications and higher mortality. If a patient’s status or the severity of the disease prohibits radical surgery, palliative operations are indicated [84,89].

Birnbaum et al. [90] performed a study in which 79% of patients presented T3 lesions, while the remaining 21% of cases had T4 lesions. An R0 resection was achieved in 86% of cases with a 3-year survival of 32%. In order to achieve R0 resection, 69% of patients required pancreatoduodenectomy, main bile duct resection, right hemicolectomy, or distal gastrectomy. Celiac or retropancreatic lymph node involvement was not considered an contraindication for curative surgery. Patients who required a pancreatoduodenectomy at 2 years after surgery had 0% survival. Similarly, patients with colonic and gastric resections had 0% survival at 3 years after surgery. Removal of other organs in addition to main bile duct resection and major liver resections proved to be a negative prognostic factor for survival.

A multicenter Dutch study [91] that included 33 patients analyzed outcomes of patients with advanced GBC who received extensive resections. All patients were submitted for main bile duct resection and lymph node dissection, with R0 resection being achieved in 16 patients. In terms of survival, 10 patients had a 2-year survival and 5 patients were alive at 5 years, with a median survival of 12. 8 months. Despite significant morbidity and mortality, 24 of the patients relapsed after a median interval of 11 months. For that reason, the authors recommend extensive surgery for carefully selected patients, weighing the risks and benefits for patients with advanced GBC.

Another retrospective study [89], including 338 patients with T3–T4 GBC, analyzed the impact of extensive surgery and negative prognostic factors on patients’ survival. They divided the 338 patients into palliative and curative surgery groups. The palliative group had 204 patients, while the curative group included 134 patients. The following rates were obtained by the authors for the curative surgical group’s survival at 3 years and 5 years: 47.3% and 44.3%, respectively. For the palliative group, patients’ survival rates at 3 and 5 years were significantly lower, at 8.3% and 7.7%, respectively. Also, for T4 patients, extended surgery provided a better prognosis, as long as R0 was achievable. The prognostic factors identified with negative impact on survival, were presence of ascites, positive margin, poor histopathological differentiation, and lymph node metastases. In conclusion, patients with advanced GBC should be evaluated carefully before determining surgical indication. As long as R0 resection can be achieved, with a good patient status, extensive surgery despite postoperative complications and mortality, brings improved survival.

### 5.5. Lymph Node Status

Lymph node status is a key step in GBC surgery, and the current AJCC edition returns to staging based on the number of examined nodes; N1 includes between 1 and 3 positive nodes, N2 ≥ 4, and a minimum of six examined nodes is mandatory. Lymph nodes are the most common site of metastasis in GBC [81], and studies show that the larger the tumor and the more structures it invades, the higher the probability of lymph node metastasis. The rate of positive lymph nodes for T1a varies between 0 and 2.5%, for T1b between 5 and 16%, for T2 between 9 and 30%, and for T3 and T4, the rate of lymph node metastasis exceeds 62.7% [81,92]. In the management of GBC, in addition to the American guidelines, which are the most widely used, the Japanese guidelines recommended by the Japanese Society of Hepato-Biliary-Pancreatic Surgery and those recommended by the Chinese Society of Biliary Surgery are also used. The guidelines provided by the Chinese Medical Association recommend examination of the pancreatic posterior nodes and para-aortic posterior nodes. If the latter is positive, then distant metastases are present and resection with curative intent is not indicated, and if the pancreatic posterior nodes are positive or ≥T3, then extensive lymph node dissection is indicated [92]. Widmann et al. [93] performed a meta-analysis in which they analyzed the impact of lymphadenectomy on patients with resected GBC with data provided and matching criteria from 18 studies. The extracted and analyzed data showed that for T1a GBC, lymphadenectomy did not improve survival compared to T1b–T3, for which lymphadenectomy influenced patients’ survival. Regarding the T4 group, the data were insufficient.

Regarding the type of lymphadenectomy mentioned in the studies, they were either specified by the number of lymph nodes obtained or by the lymph node stations, D1 and D2 lymphadenectomies. D1 lymphadenectomy includes lymph nodes of the hepato-duodenal ligament–hepatic artery and portal vein, cystic duct, and bile duct, and D2, a more extensive lymph node dissection, including celiac, para-aortic, and pancreaticoduodenal lymph nodes. The number of nodes varied between three and eight depending on how extensive the lymph node dissection was, and seven included studies showed a survival benefit and D2 lymph node dissection is recommended in advanced GBC. Tang et al. [94] showed in a study of 133 included patients that using the whole fusion lymph node dissection method improved the total number of harvested lymph nodes obtained. Therefore, en-bloc removal of lymph nodes along with surrounding fat, small vessels and nerves increased the harvested number of nodes from 6.1 ± 4.7 for the standard group to 12.00 ± 6.95 as did the number of positive lymph nodes identified, with an average of 1.85 vs. 0.78. This fact resulted in improvements in survivals and disease-free intervals compared to conventional lymph node dissection due to the fact that in using this method small nodules and small nerves and vessels that might be invaded are removed, ensuring better tumor clearance.

The lymph node ratio (LNR), which means the number of positive nodes in relation to the number of excised nodes, is another method used by researchers to improve prognostic information in patients with GBC. LNR has been shown to be an independent prognostic factor and a higher LNR indicates a worse prognosis especially for advanced GBC [3,95,96]. Total lymph node count (TLNC) is important for accurate staging. Tsimiligras et al. [97] used a machine-based approach that relies on artificial intelligence and data extracted from the National Cancer Database to find out the minimum lymph node count and range that would yield optimal results for patients with GBC. The best survival was found for patients who had between four and seven nodes resected regardless of the T, with a median survival of 40 months compared to patients who had less than four nodes and a 27% risk of death for the latter, which may suggest substaging. Also, patients who had more than three lymph node metastases in the 4–7-node group had a survival of 16.5 months compared to patients without metastases who had a survival of 90.6 months. Even so, an overwhelming proportion did not meet the AJCC recommended criteria, indicating that the number of six nodes can be challenging to achieve and 67.5% of patients have fewer than four nodes, but a minimum of four nodes are required to achieve good results. Difficulties in obtaining a TLNC ≥ 6 after portal lymphadenectomy were also encountered by Leigh et al. [98] in a retrospective analysis of ≥T1b cases. In this study, 80% of patients had fewer than six nodes identified, with an average of three positive nodes for ≥6 nodes and one positive node for the <6 nodes group. One possible explanation could be that in the hepatoduodenal ligament region there are actually fewer nodes, suggesting that to reach a number of at least six nodes, a more extensive lymphadenectomy should be performed. For that, the authors recommend including routine peripancreatic lymph node dissection for all ≥ T1b. Lymph node involvement is an important prognostic factor and the indication for the extent of lymph node dissection must be evaluated very well in order not to underdiagnose the patient. It is preferable that GBC be operated on in high-volume centers where surgeons are more experienced in hepatobiliopancreatic surgery and where pathologists are more experienced in assessing the final diagnosis and staging. Even though there are no studies that evaluate the impact of surgeon experience or the hospital’s volume on outcomes of resections performed for GBC, a study from 2020 proved that having surgery for GBC at a high-volume center is associated with a lower risk of postoperative complications and perioperative morbidity, fewer hospitalization days, and improved overall survival [99].

#### Distant Metastasis

Lately, surgical treatment for GBC with distant metastases is no longer a recommendation, rather adjuvant therapies, with an average survival of between 2 and 9 months [100,101,102] and a 5-year survival rate of 6.7–12.5% [103,104]. The reported survival for metastatic patients with GBC differs according to the site of metastasis, such that for patients with bone metastases, survival is 3 months; for those with liver metastases 4 months; 9 months for patients with lung metastases; and 6 months for patients with distant lymph node metastases, with median survival of 4 months, for the entire cohort. Improvements in survival can be achieved for patients with liver and lymph node metastases if surgery of the primary tumor is performed and, subsequently, combined with chemotherapy. For patients with bone or lung metastases, surgery is not recommended. Associated chemotherapy for patients with bone metastases can prolong survival by up to 5 months [101,105]. For patients with small liver metastases and no negative prognostic factors, such as jaundice or peritoneal dissemination, minor hepatectomy and adjuvant therapy may be a feasible treatment option. Patients who underwent T2 minor hepatectomy with less blood loss and shorter surgery times had better survival [106]. Using neoadjuvant chemotherapy, cases classified as locally advanced or metastatic and initially inoperable, after administration of different combinations of chemotherapy, patients experienced downstaging of the disease, which made R0 surgery predicable and achieve better survival [107,108,109,110,111].

### 5.6. Perineural Invasion

Perineural invasion (PNI) is an important factor that influences the prognosis and aggressiveness of malignant tumors associated with lower survival rates, and it was defined for the first time, in 1985, by Batsakis as malignant cells that invade in, around, and through nerves [112]. Multiple factors are required in order for PNI to occur. Tumor microenvironment and Schwann cells are involved in the initiation process, along with a synthesis of the specific growth and chemostatic factors. Neurotrophin factors like TGF are associated with the growth and survival of nerve cells and axon formation. Additional cell adhesion molecules enhance the cell–cell bond and cell–extracellular matrix trough receptors. These factors along with long noncoding RNA and microRNA participate in the control, regulation, and differentiation of the cell cycle [113]. To determine whether PNI is positive, the microscopic characterization should show an encircling of the nerve by tumor cells on at least 33% of the nerve circumference or malignant cells present within any nerve sheath layer [114,115,116,117]. Gallbladder and the common bile duct are surrounded by an extensive nerve network [118,119], and, therefore, PNI is frequently encountered in GBC, especially in advanced cancer, and it is one of the factors affecting the survival of patients with GBC. Few studies specifically assess PNI in GBC, and it is not included in the list of factors or potential prognostic factors, according to the AJCC’s 8th edition, and, therefore, the role of PNI in patients’ prognosis is insufficiently studied. T1 GBC tumors are almost never associated with PNI [120]; for T2 tumors, however, a meta-analysis, showed upon a sensitivity analysis, that PNI was more common in T2 peritoneal vs. T2 hepatic tumors [86], and, for stages T3–T4, PNI is more frequent. Survival for patients with PNI was 12 months vs. 34.7 months for patients without PNI [121]. Yamaguchi et al. [122] noted that PNI is an independent prognostic factor with a 5-year survival rate for patients with a PNI of 7% vs. 72% for patients without PNI, and they observed no association between PNI and lymphovascular invasion, suggesting rather that PNI occurs as a result of primary tumor extension. In terms of tumor location, PNI was higher in the center of the tumor vs. margins. An association between PNI and common bile duct invasion was also found, and where invasion is present, frozen examination of the distal common bile duct stump is recommended. Maruyama et al. [120] detected PNI as an independent prognostic factor, and PNI was positively implicated more frequently in tumors located in the liver or the neck or cystic duct, without an association with lymphatic invasion. This suggests rather different pathways of the extension of the tumor process. From the perspective of PNI, the authors showed no additional survival benefits associated with bile duct resection in terms of distal tumor location but rather for tumors located in the neck and cystic duct. Birnbaum et al. [90] identified, in a study of patients with advanced GBC, that PNI is an independent prognostic factor, and local recurrence was identified in patients with an initial PNI, with a survival of 6% at 5 years. A study from China, based on the institutions’ 10 years of experience, gathered 324 patients who underwent an operation for GBC to specifically assess the prognostic impact of PNI; 19.8% of patients were PNI-positive, with most patients being in an advanced stage of the tumor, with worse overall and disease-free survivals than patients without PNI. The median survival time was 19 vs. 45 months and median time without recurrence was 10 vs. 36 months. PNI positive patients had a lower R0 rate, was associated with preoperative jaundice, but also with a higher incidence of lymph node metastases, lymphovascular invasion, liver invasion and moderate-to-poor differentiation grade. PNI patients who received adjuvant treatment had a median survival of 24 months vs. 17 months for patients who received no treatment and a disease-free interval of 13 months vs. 6.5 months for the same group of patients. Although the impact of PNI was not very large in terms of overall survival, PNI had a large influence in terms of early relapse, and the authors’ recommendation is adjuvant therapy for PNI positive patients [123]. Therefore, according to the published results, PNI should be considered and included in the list of factors impacting the survival of patients with GBC and further studies are needed to validate PNI as a prognostic factor in GBC.

### 5.7. Lymphovascular Invasion

Lymphovascular invasion (LVI) is another risk factor associated with the poorer prognosis of patients with GBC. Few studies are specifically conducted to assess the impact in terms of survival, and most of information comes from clinicopathological descriptions made by authors in retrospective studies on GBC [120,124,125,126,127,128]. Shibata et al. [129] recommended, on the basis of the presence or absence of LVI, radical surgery for incidental gallbladder cancer, and Dominguez et al. [130] showed that LVI was present in 46.4% of 1649 patients with GBC and was the strongest predictor for the presence of lymph node metastasis, with a sensitivity of 57.1% and a specificity of 77.7%. Median survival was lower than those without LVI, for stage T1b, 26 months vs. 76.2 months; T2, 25.8 vs. 42.6 months; and T3, 11.9 months vs. 18.6 months. Further studies are needed to clearly establish the role of LVI in GBC.

### 5.8. R0 Status

R0 surgical treatment remains the only option with curative character for patients with resectable GBC. The more advanced the GBC, the more difficult it is to obtain R0 and, in some cases, multi-organ resections are required. Multi-organ resections result in the development of postoperative complications and higher morbidity. For this, experienced centers are preferred, and, clearly, R0 resections prolong survival compared to R1 resections [131]. For incidental gallbladder cancer (IGBC), in order to achieve R0, extensive oncological reoperation is needed. IGBC is a rare entity, with a frequency of 0.6% in all patients who had laparoscopic cholecystectomy [132] and is defined as GBC diagnosed histopathologically after cholecystectomy for benign gallbladder disease. IGBC should not be confused with GBC recognized intraoperatively, where curative surgery can be performed with a previously frozen examination. After histopathological diagnosis, the patient needs further investigation for correct staging and re-intervention if the tumor is ≥T1b, with the aim of achieving R0 resection [133]. Patients with IGBC have a better overall survival than patients with GBC, probably because of a less advanced T stage [132]. The time from cholecystectomy to completion of surgery with curative intent is very important, and the optimal time for reintervention is between 4 and 8 weeks. If the patient undergoes surgery earlier than 4 weeks, inflammation is present from the previous surgery and makes surgery more difficult and prone to complications, and if later than 8 weeks, the disease progresses and spreads [126]. Port site disease insemination occurs in 10%, and port site resection is not routinely recommended unless gallbladder perforation and bile leakage occurred during primary surgery [134]. Also, the histopathological analysis of the cystic duct is important. Vega et al. reported in a study that 17% of patients with IGBC had a positive margin, and main bile duct resection provided a 5-year survival of 75%, similar to patients who had a negative cystic duct margin, with a survival of 78%. Patients with a positive margin who did not have main bile duct resection had a survival of 26% [135]. Overall, whether IGBC or not, the discussion of routine main bile duct resection has been debated over time, and studies show that it brings no additional benefit [136] and retains its indication for patients with neck and cystic duct involvement [120] or patients with main bile duct invasion [137]. For carefully selected locally advanced GBC cases, major liver resection and pancreatoduodenectomy can be performed to achieve R0, even though greater complications can occur, such as liver failure, bile fistula, and pancreatic fistula; such surgery is reserved for experienced centers and offers a better survival than inoperable cases, at 25% to 5 years [138,139,140,141,142,143].

Regarding the extent of liver resection, wedge resection vs. segment IVb–V hepatectomy is still debated. A meta-analysis shows that wedge resection (1–2 cm) is sufficient as long as the margins are negative and the oncological results are comparable to those obtained in segment IVb–V hepatectomy cases. Postoperative complications were also lower with wedge resection. Indications for wedge resection remain for T2 and T3 GBC and for IGBC cases requiring reoperation, and contraindications are reserved for T1 cases, where simple cholecystectomy is sufficient, and T4 cases, where major hepatectomies are required [88]. In conclusion, in order to achieve R0, a very good preoperative evaluation, weighing the risks and benefits for the patient, for a better long-term prognosis and referring the patient to a center with large volume and experience in hepato-biliary-pancreatic surgery are necessary.

## 6. Conclusions

GBC is still associated with poor prognosis in terms of long-term survival and, given how early stages have better outcomes, early diagnosis is key for improved survival. The risk factors for GBC are divided into modifiable and nonmodifiable ones. In terms of prevention, modifiable ones, such as obesity, life-style, and diet, should be addressed. Also, procedures and programs to reduce exposure to heavy metals, AFs, and OA should be developed in specific geographical areas. Since a simple US can identify GPs, which are precursors to GBC, and gallstones, which are associated with GBC, they should be routinely performed, especially for patients predisposed to such conditions. A patient with GBC should be carefully and thoroughly evaluated before a decision is made regarding treatment options, avoiding understaging. Also, when GBC is diagnosed, it is recommended that the patient be referred to an experienced surgical center. Both the technique and experience of the surgeons, along with the pathological examination that provides the final staging, are particularly important and increase patients’ chances of survival. Neoadjuvant chemotherapy can convert an inoperable case into an operable one. In terms of prognostic factors, TNM staging, negative resection margin, and R0 status are the most important, providing a patient with GBC with the best chance of long-term survival. Also, a standardization of definitions for different terms and techniques should be applied to facilitate research work and to reach a common denominator.

## Figures and Tables

**Table 1:** The American Joint Committee on Cancer’s 8th edition of the gallbladder cancer staging system.

TNM staging	
T category	
Tis—carcinoma in situ; T1—tumor invades the lamina propria or muscularis; o T1a—limited to lamina propria; o T1b—invades muscularis; T2—tumor invades the perimuscular connective tissue; o T2a—tumor invades the perimuscular connective tissue on the side of the peritoneum; o T2b—tumor invades the perimuscular connective tissue on the side of the liver, without invading the liver; T3—tumor invades the serosa and/or directly invades the liver and/or one other adjacent structure (stomach, duodenum, colon, pancreas, omentum, or extrahepatic bile ducts); T4—tumor invades the hepatic artery, main portal vein, or two or more extrahepatic organs.	
N category:	M category: distant metastasis
N0—no regional lymph node metastasis;	M0—no distant metastasis;
N1—metastasis in 1–3 regional lymph nodes;	M1—distant metastasis.
N2—metastasis in ≥4 regional lymph nodes.	
Stages	
- Stage 0: Tis N0 M0; - Stage I: T1 N0 M0; - Stages II and IIa: T2a N0 M0; IIb: T2b N0 M0; - Stages III and IIIa: T3 N0 M0; IIIb: T1–T3 N1 M0; - Stages IV and IVa: T4 N0–N1 M0; - Stage IVb: any T, N2, M0, or any T, any N.	

## Data Availability

Data are available upon request.
